# A55 FROM FETAL REVERSION TO INTESTINAL TUMORIGENESIS: PROBING MOLECULAR REGULATORS IN THE TGFß AND HIPPO PATHWAYS

**DOI:** 10.1093/jcag/gwad061.055

**Published:** 2024-02-14

**Authors:** J Czubik, T Javkar, A Gregorieff

**Affiliations:** Anatomy & Cell Biology, McGill University, Montreal, QC, Canada; Research Institute of the McGill University Health Centre, Montreal, QC, Canada; Anatomy & Cell Biology, McGill University, Montreal, QC, Canada

## Abstract

NOT PUBLISHED AT AUTHOR’S REQUEST

Funding Agencies

None

